# Mucosal-associated invariant T cells in cancer: dual roles, complex interactions and therapeutic potential

**DOI:** 10.3389/fimmu.2024.1369236

**Published:** 2024-03-13

**Authors:** Mesut Yigit, Omer Faruk Basoglu, Derya Unutmaz

**Affiliations:** ^1^Human Immunology Laboratory, Acibadem University School of Medicine, Istanbul, Türkiye; ^2^Jackson Laboratory for Genomic Medicine, Farmington, CT, United States

**Keywords:** MAIT cell, CAR-MAIT, immunotherapy, cancer, microbiome, peripheral blood mononuclear cells

## Abstract

Mucosal-associated invariant T (MAIT) cells play diverse roles in cancer, infectious diseases, and immunotherapy. This review explores their intricate involvement in cancer, from early detection to their dual functions in promoting inflammation and mediating anti-tumor responses. Within the solid tumor microenvironment (TME), MAIT cells can acquire an ‘exhausted’ state and secrete tumor-promoting cytokines. On the other hand, MAIT cells are highly cytotoxic, and there is evidence that they may have an anti-tumor immune response. The frequency of MAIT cells and their subsets has also been shown to have prognostic value in several cancer types. Recent innovative approaches, such as programming MAIT cells with chimeric antigen receptors (CARs), provide a novel and exciting approach to utilizing these cells in cell-based cancer immunotherapy. Because MAIT cells have a restricted T cell receptor (TCR) and recognize a common antigen, this also mitigates potential graft-versus-host disease (GVHD) and opens the possibility of using allogeneic MAIT cells as off-the-shelf cell therapies in cancer. Additionally, we outline the interactions of MAIT cells with the microbiome and their critical role in infectious diseases and how this may impact the tumor responses of these cells. Understanding these complex roles can lead to novel therapeutic strategies harnessing the targeting capabilities of MAIT cells.

## 1 Introduction to MAIT cells

Mucosal-associated invariant T (MAIT) cells represent a conserved arm of the immune system, bridging the innate and adaptive responses that play a crucial role in the body’s defense against infectious diseases, particularly at mucosal sites (1–4). They are distinguished by their specialized semi-invariant T cell receptors (TCRs), primarily composed of Vα7.2-Jα33 in humans, paired with a limited set of β-chains, which recognize riboflavin (vitamin B2) metabolites, produced by bacteria and fungi, presented by the MR1 molecule on the surface of infected cells (3–7). This unique antigen recognition mechanism allows MAIT cells to respond to a wide range of microbial infections, as these metabolite antigens are conserved across many bacterial and fungal species, not found in human cells (1, 6, 8–12). MAIT cells are also involved in tissue repair and regeneration, privileged interaction between defined members of the microbiota, which sequentially controls both tissue-imprinting and subsequent responses to injury (13, 14).

**Figure 1:**
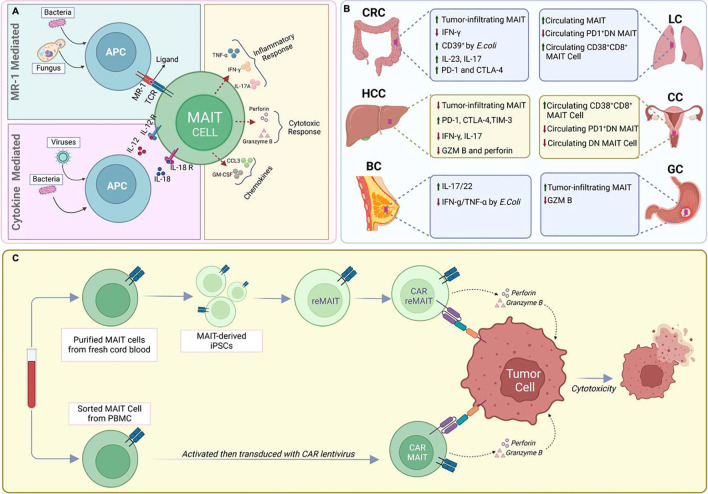
MAIT cells in infectious diseases and Cancer. **(A)**. Recognition of viruses or bacteria by MAIT cells **(B)** Functional and phenotypic features of tumor-infiltrating MAIT cells. **(C)** CAR-T cell engineering of natural or IPS-derived human MAIT cells against cancer.

Upon encountering these microbial metabolites, MAIT cells are quickly activated and initiate a potent immune response through two distinct pathways: TCR-dependent (MR1-dependent) and TCR-independent (MR1-independent) mechanisms (**Figure 1A**). In the TCR-dependent pathway, they swiftly secrete a variety of pro-inflammatory cytokines, such as IFN-γ, TNF, IL-17, and express cytotoxic molecules like granzyme B and perforin. This enables them to recruit other cells to the site of inflammation such as neutrophils and modulate others such as dendritic cells and macrophages, thus influencing the broader immune response (1, 6, 8, 15–17). Additionally, their activation is augmented by cytokines such as IL-18, IL-23, and IL-1β, as well as TLR agonists and bacterial products (16, 18–21). This multi-signal approach ensures robust MAIT cell activation and proliferation, integral to their role in the immune response.

MAIT cells can also be activated in an TCR-independent (MR1-independent) manner by cytokines released in response to viral infections or by other immune cells. IL-12 and IL-18 are key players in this cytokine-driven pathway, and additional cytokines like IL-7, IL-15, and type-I IFNs also play a role (16, 22–24). The activation mediated by cytokines could offer an additional level of protection against viral infections. This is particularly relevant because these infections do not generate the riboflavin byproducts required for activation through the MR1 pathway (16, 24).

## 2 MAIT cells during infectious diseases

MAIT cells play a crucial role in the immune response to infections (**Figure 1A**). They have been shown to directly clear multidrug-resistant bacteria and overcome mechanisms of antimicrobial resistance (25–29). Studies have also supported the protective role of MAIT cells in the antimicrobial immune response, as suggested by mouse models where the deletion of MR1, and hence MAIT cells, rendered mice more susceptible to bacterial infections (10, 30). Moreover, activated MAIT cells produce diverse cytokines and cytotoxic effector molecules and accumulate at the site of infection, demonstrating their protective role against various bacterial infections (31–33), including tuberculosis (26, 34), Salmonella (8, 35), Legionella (36, 37), Francisella tularensis (36, 38), klebsiella pneumoniae (39) and clostridium difficile (40). Zhao et al. demonstrated that systemic infection of mice with Francisella tularensis live vaccine strain (LVS) led to substantial expansion of MAIT cells in organs and blood. The study revealed a Th1-like MAIT-1 phenotype, featuring specific transcription factors and cytokine profiles, pivotal for controlling bacterial replication (41). Following infection resolution, Zhao et al. observed a transition of expanded MAIT cells into stable memory-like populations, suggesting a potential foundation for a Francisella tularensis vaccine (41).

Zheng et al. provide a comprehensive review on MAIT cells in gastrointestinal bacterial infections, revealing their intricate roles. They emphasize recent findings, showcasing the dynamic nature of MAIT cell responses, which vary based on etiological agents and anatomical locations (42). MAIT cells can also improve bacterial control during chronic infection and reduce bacterial loads through IL-17A-dependent mechanisms (26, 43).

MAIT cells have also been implicated in the immune response during viral infections such as COVID-19 and HIV infections. In the context of HIV infection, MAIT cells are depleted early on, but they retain functional cytokine expression, suggesting a potential role in controlling microbial translocation in the gut during HIV infection (34, 44). Additionally, MAIT cells have been shown to respond to and suppress HIV-1, suggesting an antiviral protective role *in vivo* (45). In the context of COVID-19, MAIT cells have been found to be highly activated and functionally impaired in COVID-19 patients and that their numbers decline sharply in the circulation (46, 47). Additionally, MAIT cell alterations markedly correlated with disease severity and patient mortality, suggesting a negative role for MAIT cells in severe COVID-19 infection, where their activation and Granzyme B production are at the highest levels (48). MAIT cells play a significant role in hepatitis infections, particularly in chronic liver diseases such as hepatitis B and hepatitis C. Studies have indicated that MAIT cells are involved in chronic hepatitis, with a decrease in their numbers independent of the cause (49). Furthermore, MAIT cells have been found to be severely reduced and exhausted in individuals with chronic hepatitis B virus (HBV) infection, suggesting a potential impact on the immune response in these patients (50)

MAIT cells were also investigated for their role in severe influenza infection. Analysis of patients with avian H7N9 influenza pneumonia revealed higher CD161+Vα7.2+ MAIT cell counts in survivors, suggesting a potential protective function. *In vitro* studies with influenza A virus (IAV)-infected lung epithelial cells and peripheral blood mononuclear cells demonstrated MAIT cell activation, characterized by upregulation of IFNγ and granzyme B. Notably, IL-18, produced by IAV-exposed CD14+ monocytes, was identified as a key factor in MAIT cell cytokine production. This IL-18-dependent activation suggests a protective role for MAIT cells in influenza and potentially in other inflammatory conditions involving IL-18 production (51).

Several authors have previously meticulously compiled comprehensive tables summarizing the multifaceted roles of MAIT cells in various infections (3, 52, 53).

## 3 Multifaceted impact of MAIT cells in cancer

MAIT cells have also been reported to serve important but diverse roles in human cancer by their dual roles in promoting inflammation and mediating anti-tumor responses, indicating their multifaceted impact on cancer (**Figure 1B**) (54–58). Associations have been determined between MAIT cell frequency, circulating inflammatory markers, and clinical parameters to elucidate the role of MAIT cells in inflammation-driven cancer, indicating their potential relevance in cancer progression (59, 60).

In multiple cancers, including glioma and others, Kubica et al. found that MR1 is overexpressed, yet its impact on overall survival varies (61). Specifically, in glioma, high MR1 levels correlate with poorer outcomes (61). However, the link between MR1 expression and survival is complex and not universally clear across cancer types since he correlation between MR1 levels and MAIT cell infiltration differs among cancers (52). Beyond solid tumors, MR1 expression is also observed in multiple myeloma cell lines, which can present the vitamin-B derivative ligand to MAIT cells, which in turn can induce cytotoxic activity towards these myeloma cells (62).

The ability of MAIT cells to exert cytotoxic effects and secrete inflammatory cytokines situates them at a crossroads of tumor biology, where they can potentially influence tumor growth, progression, and response to treatment (55, 58, 63, 64). However, the role of MAIT cells in cancer is not straightforward (52). Petley et al. showed that MAIT cells enhance NK cell mediated anti-tumor immunity by inducing an IFN- γ transcriptome in NK cells followed by activation (58). As such, it is worth noting that MAIT cells can play a supplementary rather than a primary role in NK cell-mediated anti-tumor activity. Additionally, Gentles et al. demonstrated that more CD8+CD161+ T cells, including MAIT cells, in tumors are linked to better outcomes in various cancers, suggesting a potential role for these cells in clinical benefits, supporting the theme of anti-tumor effects (65). Additionally, MAIT cells have been shown to influence the tumor microenvironment and could be relevant in cancer immunotherapy, although their exact role remains uncertain (57, 58, 63).

Conversely, other studies have highlighted the tumor-promoting functions of MAIT cells. For example, they can suppress T and NK cells and blocking the MR1 protein may offer a new strategy for cancer immunotherapy (57). In non-small cell lung cancer patients, MAIT cells exhibited an exhausted tumor-promoting phenotype and were found to predict the response to anti-PD-1 immunotherapy (66). Negative impact of IL-17 producing MAIT cells on cancer progression has also been observed, suggesting a potential detrimental role in cancers such as breast cancer (63). MAIT cells can also impair anti-tumor immunity by expressing PD-1 ligands, which can in turn lead to conventional T cell exhaustion and reduced anti-tumor responses in prostate cancer (67).

The paradoxical behavior of MAIT cells that encompasses both the promotion of tumor growth and the execution of anti-tumor activities is possibly result of their ability to secrete a wide range of cytokines and cytotoxic molecules, influenced by the signals they receive from the tumor microenvironment (TME) (57–59, 68–71). On the one hand, MAIT cells have been observed to produce pro-inflammatory cytokines such as IL-17 and IL-13 within the TME (59, 72, 73). IL-17 is known to be involved in chronic inflammation and has been linked to the promotion of angiogenesis and tumor growth (74, 75). It can enhance the survival and proliferation of cancer cells, and its presence in the TME is often associated with a poor prognosis in several cancers (74–77) Similarly, IL-13 has been implicated in tumor progression and metastasis (73, 78). It contributes to an immunosuppressive TME by inhibiting the activity of cytotoxic cells and promoting the survival and proliferation of tumor cells (73, 78).

MAIT cells are also primarily found in mucosal tissues such as the gut, lungs, and cervix, and are thought to play a crucial role in the immune response in these tissues. As such, they could particularly migrate into tumor areas within these areas, including colorectal and cervical cancers (79). Their prevalence in these tissues positions them as potential early detectors and responders in the development of cancer, serving as the first line of defense against abnormal cellular changes that may lead to cancer (79).

The potential migration of MAIT cells into tumor areas within mucosal tissues has also been supported by the observation that circulating MAIT cells are reduced in mucosa-associated cancer patients, possibly because of migration to these areas (68). MAIT cells were also shown to accumulate in colon adenocarcinomas, indicating their potential to promote local immune responses to tumors, although factors in the tumor microenvironment may act to reduce MAIT cell IFN-γ production (79). Moreover, MAIT cells have been found to display hallmarks of bacterial antigen recognition in colorectal cancer (56). It was also suggested that MAIT cells in colorectal cancer, when activated, can be potential sources of stimulation in the tumor microenvironment (55).

In cervical cancer there also seems to be a link between the number of MAIT cells and myeloid-derived suppressor cells (MDSCs). This connection suggests that MAIT cells, through the release of IL-17 and other cytokines, could be involved in recruiting MDSCs to the tumor site, potentially aiding in tumor growth and progression (54; Lu et al., 2020). Increased CD8+, CD4+, and highly activated CD38+CD8+MAIT cells in cervical cancer patients’ peripheral blood compared to healthy donors, along with reduced PD1+ double negative (DN) MAIT cells were also observed (Lu et al., 2020). Importantly, higher levels of circulating PD1+ DN MAIT cells correlated with improved progression-free survival in cervical cancer patients (Lu et al., 2020).

Several studies that have also focused on MAIT cell frequencies as prognostic factors in these cancers. In colorectal cancer (CRC) it was observed that MAIT cell numbers in the periphery decrease, possibly because these cells migrate preferentially to the cancerous areas (59, 70). This pattern of MAIT cell redistribution has also been observed in patients with gastric cancer (GC) and cervical cancer, indicating a similar trend in these types of cancer as well (64, 80). Whereas, increased circulating MAIT cells appear to be correlated with lung cancer progression, further suggesting their potential role in tumor areas within the lungs (71). To provide a concise overview of changes in MAIT cell function across various solid tumors, a summary is presented in **Table 1**. These clinical observations highlight the potential prognostic potential of MAIT cells in cancer.

**Table 1:** MAIT cells in human cancer.

Human cancer Type	Functionality Changes in response to TME	Reference/Year Published
Colorectal Cancer (CRC)	**↑**Tumor-infiltrating MAIT **↑**CD39+ MAIT cells (stimulated by E. coli) **↑**PD-1 and CTLA-4 **↑**Ki-67 ↓IFN-γ	(56)
	**↑** IL-23 **↑** IL-17 **↑** IL-6	(81)
	**↑**Tumor-infiltrating MAIT **↑**IL-17 secretion from MAIT	(82)
	**↑**Tumor-infiltrating MAIT ↓IFN-γ producing MAITs No change in TNF-α or IL-17	(79)
	**↑**Tumor-infiltrating MAIT ↓IFN-γ **↑**IL-17A	(59)
	**↑**Tumor-infiltrating MAIT ↓IFN-γ	(83)
	**↑**Tumor-infiltrating DN MAIT **↑**PD-1^high^Tim3^+^CD39^+^ exhausted phenotype	(70)
Prostate cancer (PC)	**↑**PD-L1/L2 upregulation on Tumor-infiltrating MAIT (induced by 5-A-RU)	(67)
Gastric cancer (GC)	**↑**Tumor-infiltrating MAIT ↓ GZM B	(64)
Cervical Cancer (CC)	↓ Circulating MAIT	(80)
	**↑**Circulating CD38^+^CD8^+^MAIT cell ↓Circulating PD1^+^DN MAIT ↓DN MAIT cell	(84)
Multiple Myeloma (MM)	↓Circulating MAIT ↓CD27 expression on MAIT ↓IFN-γ production from MAIT	(62)
Breast carcinoma	**↑**IL-17/22 ↓IFN-γ/TNF-alpha by E. Coli	(85)
Colorectal liver metastases (CRLM)	↓Tumor-infiltrating MAIT ↓IFN-γ	(72)
Hepatocellular carcinoma(HCC)	↓Tumor-infiltrating MAIT	(86)
	↓Tumor-infiltrating MAIT **↑**PD-1, CTLA-4, TIM-3 ↓IFN-γ, IL-17, GZM B and perforin	(87)
Lung Cancer (LC)	**↑**Circulating MAIT ↓Circulating PD1^+^DN MAIT **↑**CD38^+^CD8^+^MAIT cell	(71)

Overall, these findings collectively suggest that MAIT cells can display contrasting roles in controlling anti-tumor immune responses depending on their activation status, may have complex and diverse roles in cancer progression and potential as therapeutic targets in cancer treatment (58). However, their exact impact on tumor progression and therapy remains a complex and evolving area of study.

## 4 Impact of tumor microenvironment on MAIT cell immune responses

As discussed above, the signals received by MAIT cells, such as cytokines, cell-cell interactions, and metabolic cues, can sway them towards either promoting tumor growth or exerting anti-tumor effects (**Figure 1B**). Understanding these signals and the conditions under which they operate is crucial for harnessing the therapeutic potential of MAIT cells in cancer (25, 56, 67, 68, 70, 73, 87).

Within the TME, MAIT cells can undergo significant phenotypic changes that alter their functionality. One of the most critical transformations is the development of an ‘exhausted’ phenotype. This state of exhaustion is characterized by a reduced capacity to proliferate, produce cytokines, and mediate cytotoxicity, alongside an increased expression of inhibitory receptors such as PD-1, CTLA-4, and TIM-3. Such phenotypic changes indicate that MAIT cells may contribute to an immunosuppressive tumor microenvironment, potentially aiding in cancer progression (25, 56, 67, 70, 87).

The path to exhaustion typically begins with chronic antigen stimulation and persistent inflammation within the TME (59, 70). As MAIT cells encounter an ongoing barrage of signals - from cancer cell antigens presented by MR1 to inflammatory cytokines and chemokines - they initially respond robustly. However, this sustained activation can lead to a state of overstimulation, may lead to the previously mentioned functional dysregulation (25, 56, 67, 70, 87).

The implications of MAIT cell exhaustion for cancer progression are manifold. Exhausted MAIT cells may contribute to an immunosuppressive TME, allowing cancer cells to evade immune detection and destruction. They may also promote the recruitment and function of regulatory cells, such as regulatory T cells (Tregs) and myeloid-derived suppressor cells (MDSCs), which further inhibit the anti-tumor response. In addition, these exhausted MAIT cells can secrete cytokines that support tumor survival, angiogenesis, and metastasis, thus inadvertently aiding cancer progression.

Furthermore, the exhausted phenotype is often associated with a reduced production of IFN-γ, IL-17, granzyme B, and perforin by MAIT cells (88, 89). This not only diminishes the direct anti-tumor activity of MAIT cells but also impacts the broader anti-tumor immune response, as these molecules play crucial roles in orchestrating and executing effective anti-cancer immunity (82, 83, 87–89).

In hepatocellular carcinoma (HCC), tumor tissue localized MAIT cells also exhibited exhausted phenotype with upregulation of inhibitory immune molecules (PD-1, CTLA-4, and TIM-3) and secreted lower quantities of effector molecules (e.g., IFN-g, granzyme B, and perforin) (87). As suggested by this immunosuppressive phenotype, high MAIT cell infiltration into HCC solid tumors was correlated with adverse prognosis (87). Furthermore, these cells often express pro-tumor cytokines like IL-8, possibly contributing to the recruitment of immunosuppressive MDSCs to the tumor site. The presence of such immunosuppressive MAIT cells in HCC has been associated with a negative impact on patient prognosis (87, 90).

Thus, the presence, functional state, and interactions of MAIT cells with other immune cells within the TME can provide valuable insights into disease progression and patient outcomes. However, the dualistic nature of MAIT cells and their varying roles in different cancer types or even within the same cancer type but at different stages or different patients underscore the need for a deeper understanding of their biology. Understanding these mechanisms is crucial because it could lead to novel therapeutic approaches that leverage MAIT cells’ natural targeting abilities including genetically engineering MAIT cells to enhance cancer treatment.

## 5 Role of MAIT cells in cancer treatment

The role of MAIT cells in positive contribution to anti-tumor immune responses is also an area of ongoing investigation (66) especially against solid tumors (65, 91). MAIT cells are capable of exerting anti-tumor effects, predominantly through the action of cytotoxic molecules (92). They can also release granzyme B and perforin, which are essential for the direct killing of target cells (23, 93). The anti-tumor activity of MAIT cells is further enhanced by their ability to produce IFN-γ, a cytokine critical for immune surveillance and the activation of other immune cells, including macrophages and dendritic cells. IFN-γ can enhance the antigen-presenting capabilities of these cells, leading to a more effective anti-tumor immune response (23, 32, 94, 95).

As discussed above, MAIT cells express immune checkpoint receptors and possess cytotoxic functions, making them attractive in tumor immunology research. Innate-like T cell subsets, including MAIT cells, have shown potential in targeting the immunosuppressive TME composed of tumor-associated macrophages (TAMs) and MDSCs (32, 63, 70). Interestingly, Biasi et al. (2021) found that detecting circulating MAIT cells could identify patients’ positive responses to anti-PD1 therapy, suggesting their promise as a biomarker for treatment effectiveness (96). Additionally, the frequency of circulating MAIT cells was observed to influence treatment response and survival in melanoma patients undergoing anti-PD1 therapy, emphasizing a potential role for MAIT cells in shaping treatment outcomes (97).

While the current understanding of how MAIT cells are activated within tumors is predominantly focused on their response to microbial metabolites (59) the possibility that they could also be triggered by antigens originating from the tumor itself has not been ruled out (55, 63). If such tumor-derived antigens that activate MAIT cells are identified, they could serve as precise targets for new immunotherapies, potentially leading to more effective cancer treatments.

Besides solid tumors, the involvement of mucosa-associated invariant T (MAIT) cells in hematological malignancies and transplantation immunity has also been a subject of interest in recent research. MAIT cells may also play multifunction role in hematological malignancies and transplantation immunity, highlighting their potential role as an immunomodulatory factor (98).

However, experimental evidence indicates that MAIT cells can retain their anti-tumor functions even when MR1 is knocked out from the tumor cells, suggesting alternative pathways through which MAIT cells can exert their effects. The nuances of MR1 expression and the conditions that influence the anti-tumor activities of MAIT cells are still under investigation. Discrepancies in MR1 expression across various tumor cell lines and the resulting differences in MAIT cell response underscore the complexity of the immune system’s interaction with cancer. Utilization of 3D bioprinted tumor models to study the effect of localization and activation of MAIT cells within the 3D tumor tissue model could help elucidate and these complex interactions (99).

Priya and Brutkiewicz et al. use an artificial antigen-presenting cell (aAPC) comprised of latex beads coated with an MR1 tetramer complex loaded with antigen (Ag) and anti-CD28 antibody, they successfully addressed MAIT cell expansion limitations (100). Furthermore, they demonstrated that aAPC-expanded MAIT cells retained functionality, preserved their original phenotype, and displayed both proinflammatory cytokine secretion and cytotoxicity against GBM cell lines (100). These findings strongly support the potential of aAPC-generated MAIT cells as a promising and efficient option for adoptive immunotherapy against tumors (100).

Recent advancements in immunotherapy have highlighted chimeric antigen receptor (CAR)-based therapies, with CAR-T cells showing promise in targeting hematological malignancies (**Figure 1C**) (52, 101). However, these therapies have significant limitations, such as severe side effects, and are less effective against solid tumors. Additionally, CAR-T therapies require autologous cells, which are costly and time-consuming to produce (102).

Utilizing MAIT cells engineered with CAR molecules (CAR-MAIT) could be a novel approach that may address some of these challenges. Recently, we developed such CAR-MAIT cells to target lymphomas and breast cancer cells (101). We had found that these engineered cells demonstrate robust cytotoxicity against CD19+ lymphomas and Her2-expressing breast cancer cell lines, while releasing lower levels of inflammatory cytokines, potentially leading to fewer adverse reactions like cytokine release syndrome (101). This approach also avoids the MHC class I restriction, enabling the development of ‘off-the-shelf’ therapies. Importantly, we were able to expand large numbers of these MAIT cells from PBMCs of healthy donors (typically more than 100-fold). Given that MAIT cell proportions can be as high as 10-20% of CD8 T cells in some donors, we believe that generating abundant cells for off-the-shelf therapeutic applications is likely achievable (101). Furthermore, MAIT cells may continue to expand *in vivo*, as they will still recognize and be activated by their common cognate antigen, thus requiring fewer cell transfers to patients.

Efforts to address the suboptimal ex vivo expansion efficiency of Mucosal-Associated Invariant T (MAIT) cells from healthy donor peripheral blood led to the development of pluripotent stem cell (PSC)-derived MAIT-like cells (**Figure 1C**) (103). Wakao et al. pioneered the reprogramming of cord blood (CB) MAIT cells into induced pluripotent stem cells (iPSCs) using a Sendai viral vector (103). The resulting MAIT-iPSC clones demonstrated pluripotency through various tests and were differentiated into MAIT-like lymphocytes using a two-step protocol (103). More recently, it was proposed that iPSC-derived Mucosal-Associated Invariant T (MAIT) cells in tumor immune cell therapy or prophylaxis may prevent relapse (104). While phenotypic and functional characteristics of reprogrammed MAIT cells do not fully mirror those of PBMC-derived MAIT cells (103), this innovative approach further aims to address the limitations associated with the ex vivo expansion of MAIT cells, such as the restricted numbers obtained after expansion compared to the ex vivo expansion of all T cells. Together, the ability to efficiently expand MAIT cells ex vivo while maintaining a functional phenotype may facilitate the development of new MAIT cell–based tumor immunotherapies.

One distinguishing characteristic that significantly contributes to their therapeutic appeal is their incapacity to induce graft-versus-host disease (GvHD) (105–107). This is attributed to their restriction by MR1 and their non-recognition of mismatched major histocompatibility complex (MHC) molecules and protein autoantigens (105). Rigorous assessments, including *in vitro* studies and xenogeneic GvHD mouse models, consistently affirm the GvHD-free safety profile of MAIT cells (108). Recent research endeavors have shed light on the multifaceted advantages of MAIT cells, extending beyond their GvHD-free safety profile. Notably, MAIT cells exhibit resistance to xenobiotics, adding an additional layer of appeal for their therapeutic application (2). Studies conducted by Bohineust et al. showcase that these engineered MAIT cells can engraft without eliciting GvHD in preclinical immunodeficient mouse models, presenting a distinct advantage over CD19-targeted CAR-T cells (109).

Furthermore, several studies demonstrated the potential for MAIT cells in solid tumor-targeting immunotherapies due to their natural ability to infiltrate tissue sites of chronic inflammation and their abundance in the tumor microenvironment (99, 101, 109, 110). In this tumor environment, MAIT cells can also act as innate T cells with the potential to bridge innate and adaptive immune immunity, and can rapidly exert their effector functions upon activation without the requirement for clonal expansion, similar to innate immune (111) (112). Upon activation, MAIT cells rapidly secrete cytokines and exert cytotoxic functions, making them highly relevant in tumor immunity (70). Indeed, such feature would be significant in that even in the case where tumors escape the attack from CAR-MAIT cells, they can still exert anti-tumor activity.

Overall, we think CAR-MAIT cells represent a significant advancement in our arsenal of immunotherapy. Their development could overcome the limitations of current CAR-T therapies and harness the immune system’s power more safely and effectively. Future studies, especially *in vivo* experiments will be needed to fully understand the therapeutic potential of CAR-MAIT cells.

## 6 MAIT cell and microbiome interplay in cancer

MAIT cells also play a crucial role in interacting with the microbiota and its products, protecting body barriers, and maintaining tissue homeostasis (113). MAIT cells have been found to be enriched in the human liver, which is constantly exposed to bacterial products from the intestine, indicating their role in the gut microbiota-liver axis (114, 115). Furthermore, MAIT cells have been shown to control barrier function and attenuate pathogenic T cell responses in the colon, suggesting their importance in maintaining gut integrity (116). The gut microbiome is crucial for the development and function of MAIT cells, and their association with the tumor microbiome supports the interest in manipulating the gut microbiome to reshape their response (56, 117).

Additionally, MAIT cells are thought to play a role in bacterial immunity, as they interact with metabolites synthesized by various yeast and bacteria (118). Moreover, MAIT cells have been proposed to play an important role in protecting mucosal surfaces against multiple bacterial pathogens (119). In contrast, studies have also revealed a deleterious role of MAIT cells in promoting inflammation, gut mucosa dysfunction, and gut microbiota alteration in obesity, indicating a complex role of MAIT cells in different contexts (3). Furthermore, the reduction and functional immaturity of MAIT cells in the peripheral blood of patients with primary Sjögren’s syndrome suggest a potential link between MAIT cells and autoimmune conditions (119).

The potential role of MAIT cells in the regulation of metabolic pathways and their crosstalk with the microbiota represents an exciting new line of research (120). In our studies focusing on activation of MAIT cells differing in their TCR Vbeta regions, we found that MAIT cells responded selectively to microorganisms that produce riboflavin metabolites, which are common across a variety of bacterial species within the microbiota (11). This suggests that MAIT cells play a role in fine-tuning the immune response to a broad spectrum of microbial inhabitants in the human body, including both commensal and pathogenic species. This ability to discriminate between different bacteria can be crucial for maintaining a balanced microbiome. The insights into how MAIT cells interact with the microbiome could have implications for understanding and treating diseases that are influenced by microbiota imbalances, such as inflammatory bowel disease, obesity, and certain types of cancer or immunotherapy as discussed below.

Growing evidence highlights a relationship between the human microbiome and cancer, particularly in the context of solid tumors. The composition of the microbiome has been found to offer prognostic insights and is implicated in the initiation, growth, and spread of tumors. Additionally, certain microbial signatures have been identified that may either contribute to tumor development or enhance the efficacy of immunotherapies (121–123).

MAIT cells, which have an affinity for microbe-rich environments and respond to microbial metabolites such as those from riboflavin metabolism, are poised to play a crucial role in the dialogue between the microbiome and the tumor microenvironment (52). They possess the unique ability to detect these metabolites presented by the MR1 protein on the surface of various cells, including tumor cells (6). Notably, Liu and Brutkiewicz et al. investigated the role of TLR9 activation in MR1-mediated bacterial antigen presentation and concluded that the activation of Toll-like Receptor 9 (TLR9) is crucial for Major Histocompatibility Complex Class I-Related gene (MR1)-mediated bacterial antigen presentation (124). Interestingly, studies have shown that melanoma cells can increase their expression of MR1 when exposed to the microbial metabolite 5-OP-RU, highlighting the potential for microbial components to modulate immune recognition (62).

Changes in the microbiota, termed dysbiosis, have been linked to tumor development and progression. Dysbiosis can lead to altered production of microbial metabolites, affecting the activation and function of MAIT cells. For example, a microbiota that favors inflammation may promote the secretion of pro-tumorigenic cytokines by MAIT cells, such as IL-17 and IL-13, contributing to tumor growth and progression (123). Conversely, certain microbial populations might induce anti-tumor responses by MAIT cells, enhancing their cytotoxic functions and the production of IFN-γ, which could suppress tumor growth. For instance, some studies suggest that specific bacterial strains can boost the efficacy of cancer immunotherapies, potentially through the activation of MAIT cells (125).

Moreover, the TME itself can alter the local microbiota, creating a feedback loop that further influences MAIT cell function (14, 126, 127). Tumor-induced changes in the local environment, such as hypoxia or altered nutrient availability, can select for microbial populations that either exacerbate or mitigate tumor progression (127).

The microbiota also plays a role in the modulation of the immune checkpoint molecules on MAIT cells. For example, certain bacteria might influence the expression of PD-1 on MAIT cells, affecting their state of exhaustion and ability to fight cancer cells (70, 128). Thus, given the central role of the microbiota in regulating immune responses, therapeutic strategies are being considered that could modulate the microbiota to enhance the anti-tumor activity of MAIT cells. These include the use of prebiotics, probiotics, or fecal microbiota transplants to reshape the microbial ecosystem in favor of a more robust anti-tumor immune response.

MAIT cells’ interactions with both cancer cells and the microbiome are integral to their dual role in tumor biology. Their capacity to respond to early cancerous changes, influenced by shifts in the microbiome, along with the ongoing research into their tumor recognition mechanisms, underscores the potential of MAIT cells as targets for innovative cancer therapies (56, 63).

## 7 Conclusions and future directions

In conclusion, current advances in MAIT cell immunobiology underscores the necessity to unravel the involvement and therapeutic potential of MAIT cells in in cancer biology, as well as to exploit the microbiome-MAIT cell interaction for the development of new cancer immunotherapies. The abundance of MAIT cells, ability to expand them *in vitro* with their antigen and possibility of generating these cells from iPSCs in humans presents an exciting avenue for future studies to develop them into CAR-MAIT cells. Other diverse avenues for future studies may include the exploration and development of immunotherapeutic strategies through utilization of MAIT cell anti-tumor recognition and functions and the potential use of small molecule or microbiome-derived therapeutics to stimulate their responses. These approaches highlight the promising prospects for advancing cancer treatment through understanding and developing MAIT cell-based therapeutics.

## Author contributions

DU: Conceptualization, Writing – original draft, Writing – review & editing. MY: Writing – original draft, Writing – review & editing. OB: Visualization, Writing – review & editing.
